# Efficiency of nitrogen, gibberellic acid and potassium on canola production under sub-tropical regions of Pakistan

**DOI:** 10.1038/s41598-023-38997-8

**Published:** 2023-10-31

**Authors:** Muhammad Mahran Aslam, Fozia Farhat, Saman Zulfiqar, Mohammad Aquil Siddiqui, Muhammad Asim, Mahboob Ali Sial

**Affiliations:** 1 Nuclear Institute of Agriculture (NIA), Tando Jam, 70060 Sindh Pakistan; 2 Department of Botany, Government College Women University, Faisalabad, Pakistan; 3 Department of Botany, The Government Sadiq College Women University, Bahawalpur, Pakistan; 4 Plant Science Division, Pakistan Agricultural Research Council (PARC), Islamabad, Pakistan

**Keywords:** Plant breeding, Plant hormones, Plant physiology

## Abstract

The global demand for crop production is rapidly growing due to the continued rise in world population. Crop productivity varies generally with soil nutrient profile and climate. The optimal use of fertilizers might help to attain higher crop yield in canola. To circumvent nutrient imbalance issues in soil, two separate field trials were conducted to determine (a) the best source of nitrogen (N) between ammonium sulfate (NH_4_)_2_SO_4_) and ammonium nitrate (NH_4_NO_3_), (b) significance of gibberellic acid (GA_3_) and potassium (K), in an attempt to enhance canola yield and yield attributes. Both experiments were carried out in randomized complete block design (RCBD) with three replicates. The nitrogen source in the form of NH_4_)_2_SO_4_ (0, 10, 20 and 30 kg/ha) and NH_4_NO_3_ (0, 50, 75 and 100 kg/ha) was applied in the rhizosphere after 3 and 7 weeks of sowing, referred to as experiment 1 (E1). In another separate experiment (E2), the canola crop was sprayed with four level of GA_3_ (0, 10, 15, 30 g/ha) and K (0, 2.5, 3.5, 6 g/ha) individually or in combination by using hydraulic spryer, 30 days after sowing (DAS). The data was collected at different growth stages of canola and analyzed statistically. The E1 trail showed that N fortification in the form of NH_4_NO_3_ (100 kg/ha) and (NH_4_)_2_SO_4_ (30 kg/ha) had a positive effect on the plant height, number of branches, fruiting zone, seed yield per plant, seed yield per hectare of canola except oil percentage. Moreover, canola plants (E2) also displayed a significant improvement on all studied features with high doses of GA_3_ (30 g/ha) and K (6 g/ha) individualy and in combined form. The correlation coefficient analysis of (NH_4_)_2_SO_4_ and NH_4_NO_3_ was highly significant to plant height, number of branches, fruiting zone, seed yield per plant, seed yield per hectare of canola In a nutshell, compared to both source of N, NH_4_NO_3_ was more efficient and readily available source of N. GA_3_ being a growth elicitor and potassium as a micronutrient serve as potential source to improve yield and to manage nutrient profile of canola.

## Introduction

For several years now, arid and semi-arid areas located in certain third world countries have been facing massive shortage of edible oils which was met through imports in large quantities from other countries^1^. As a result, efforts aiming at reducing the imbalance between the production and consumption for edible oils have been made by under-developed countries. In this context, oil seed crops seemed to be an accurate option for these countries. Among these crops, canola appeared as a potential candidate for the domestic edible oil production^2^. This could be explained by to the low content of erucic acid and glucosinolates in oil and its seed cake, respectively^3^. Moreover, canola crop can survive under diverse environmental conditions due to a wide range of adaptability^4^. However, mismanagement and highly imbalanced application of micro and macronutrients found to be reducing the yield of canola crop, therefore, nutrients management strategies for optimizing the canola production are highly required^5^.

Balanced fertilizer application influence the crop yield, quality and the soil productivity^6^. The adequate nitrogen supply is important in order to boost up the canola productivity and it holds a key role in plant tissue growth and development. Plus, it represents a part of chlorophyll, nucleotides, protein, and amino acids formation which directly affect the quality and quantitative traits of the crop. Other factors such as Soil profile, texture, and moisture content fluctuation at various critical stages of growth and development of canola may influence the nitrogen use efficiency on canola crop. Actually, this kind of crop can very responsive to fertilizer application, especially nitrogen which significantly impacts the plant height, number of branches/plant, number of flower/plant, number of pods/plant and their weights, and seed yield/ha. It also effects of the leaf area (LA) development and LA duration after flowering in canola crop^7,8^.

Many natural and artificial plant regulators may be used with the aim of controlling the developmental process from germination to post-harvest preservation of crop plants and subsequently, optimizing their production^9^. Among these fertilizers, gibberellic acid (GA_3_) is obviously a key regulator product for plant-growth and other physiological mechanisms. It can stimulates the root and stem elongation, seed germination, break dormancy, leaf expansion, fruit senescence, and flowering^10^. Moreover, GAs may influence the metabolic pathways including nitrogen metabolism, chlorophyll production and degradation, nitrogen redistribution, and translocation of assimilates^11^. It can also induces the expression of several hydrolytic enzymes involved in the conversion of starch to sugar which ultimately influence the plant growth at vegetative and reproductive stages^12^, plant signaling mechanisms, gene expression, and plant morphology and physiology^13,14^.

Besides nitrogen and phosphorus fertilizers, K found to be influencing the seed oil content percentage, yield and yield-contributing traits of the canola crop^15,16^. K is very important fertilizer which is involved in photosynthesis, regulation of stomata, control of the ionic balance, translocation of photosynthates, protein synthesis, enzymatic activities, and many other physiological and biochemical processes^17,18^. Therefore, K is considered as primary osmoticum that plays an important role at maintaining the low water potential in plant tissue and also impacts the plant growth and development.

For plant breeders, the efficient use of nutrients from the soil by the crop plants is a promising characteristic. Some plants may produce high yields with minimal inputs^14,18^. Many studies showed that significant variation exists among various genotypes of canola regarding efficient use of potassium^19^.

Keeping in view the possible outcomes of efficient use of K and GA, the current study evaluates canola genotype (Surhan-2012) for four consecutive years for these traits. Hence, current manuscript demonstrated the influence of foliar application of GA_3_ and K separately, or in combination in canola. This study carries immense importance as a reference for the impact of these two important nutrients on canola production and the multi-year screening of Surhan-2012 in this context. The main objective of the study to find out the best source of nitrogen and optimal combination of GA_3_ and K for increasing the canola productivity. Being major oil importer country, overall objective to introduce cost effective technique to enhance canola production.

## Material and methods

### Site descriptions

The two field experiment was conducted at the agriculture farm of Nuclear Institute of Agriculture (NIA), Tando Jam, Sind, Pakistan (31° 25′ 0″ North, 73° 5′ 0″ East) and an altitude 30 m above from sea level. The experimental farm was irrigated by the canal water from the river Sindh. The experiment 1 (E1) trial was conducted at two growth seasons (2017–18/2018–19) while experiment 2 (E2) trial was managed for four consecutive years (2014–18). The physical and chemical properties of the soil at the study site are presented in Tables 1 and 2. This was carried out to ascertain the characteristics of the soil at the experimental site. The soil test result obtained showed that the soil was sandy loam and pH (7–7.5). The detail status of soil agronomy and characteristics was depicted in (Table 1). The experimental area is semiarid with very low rain fall (100–300 mm, the about 70% rainfall occurs in summer and the remaining 30% occurs in winter season.

**Table 1:** Details status of soil agronomy research field.

NIA exp. field										
pH	OM (%)	Total N (%)	(meq/60 g soil) (μg/g soil)							
			Na	K	Mg	P	S	Ca	Zn	B
7.0–7.5	1.05	0.07	0.07	0.12	2.1	12.3	16.09	3.02	1.43	0.38

Source: Soil Science department (2018).

**Table 2:** Treatment levels/doses of potassium (K) and gibberellic acid (GA_3_) on canola production.

Treatments	Potassium-K (g/m^2^)	Gibberellic acid (g/ha)
T1	0 (control)	0 (control)
T2	2.0	0
T3	3.5	0
T4	6.0	0
T5	0	100
T6	0	150
T7	0	300
T8	2.0	100
T9	3.5	150
T10	6.0	300

### Experimental design

Two field experiments of canola were designed to conduct at the farm of Nuclear Institute of Agriculture (NIA), Tando Jam, Sind, Pakistan (31° 25′ 0″ North, 73° 5′ 0″ East). The experiment dealing with ammonium sulfate (NH_4_)_2_SO_4_ and ammonium nitrate NH_4_NO_3_ designated as experiment 1 (E1) to counter best source of N supplementation and application of K and GA_3_ referred to as experiment 2 (E2). The E1 trial was conducted at two growth seasons (2017–18/2018–19) while E2 trial was managed for four consecutive years (2014–18). Data were collected under a randomized Complete Block Design (RCBD) with three replications per block. The both experiments was sown in winter season (September–March) during all growing seasons.

The canola seeds were collected from nuclear institute of agriculture and sown and thinned after 15–20 days of germination for the purposes of maintaining long distance dispersal of plants. The plant to plant and row to row distance was maintained 9 and 18 in., respectively. All the recommended agronomic and cultural practices that govern the production of the crop were applied efficiently during the plant growth cycle^20^.

### Application of (NH_4_)_2_SO_4_ NH_4_NO_3_ as N supplements

The four levels of (NH_4_)_2_SO_4_ (0, 10, 20 and 30 kg/ha) and NH_4_NO_3_ (0, 50, 75 and 100 kg/ha) were used as nitrogen source. The (NH_4_)_2_SO_4_ composed of 21% nitrogen and 24% sulfur and NH_4_NO_3_ contained 33.5% nitrogen. Both nitrogen fertilizers were applied in two split doses; the first dose was applied after 3 weeks of crop sowing whereas the second was undertaken after 7 weeks of sowing. One square meter (m^2^) area of plants was chosen randomly from each plot for harvesting during two seasons (2017–18/2018–19). The agronomic parameters of crops were computed from the plant height (cm), number of branches/plant, fruiting zone length (cm), seed yield/plant (g), seed yield/ha (kg). The differences of oil content (%) of canola seeds were recorded, pooled and statistically analyzed in order to evaluate the effect of different sources/doses of nitrogen on the agronomic characters and traits of canola^16^.

### The effect of K nitrate and GA_3_ on canola yield and yield attributes

Ten different combinations (Table 2) of K and GA_3_ were applied as foliar spray. The experiment was carried out using a randomized complete block design (RCBD) with three replications.

Before the foliar application, GA_3_ was dissolved in ethanol. Various dilutions were then made in order to obtain solutions with several concentrations. The different combinations of GA_3_ and K were sprayed after 1 month of sowing. The treatments were applied three times with an interval of 1 week and the control plants were sprayed with distilled water only. One m^2^ area of plants was chosen randomly from each plot at harvesting time during four seasons (2014–18). The data of agronomic parameters including Plant height (cm), number of branches/plant, fruiting zone length (cm), seed yield/plant (g), seed yield/ha (kg) and oil content percentage have been recorded according to the protocol reported by A.O.A.C in 1980. Subsequently, the recorded data were analyzed using analysis of variance (ANOVA) combined with HSD. Tukey’s test was also used to determine the significant difference between the treatments with the help of statistical software SAS (version 9.4) and finally calculation of the cost–benefit ratio.

### Ethical approval

All the plant studies were carried out in accordance with relevant guidelines and regulations of concern Institute (Nuclear Institute of Agriculture (NIA) Tandojam Sindh Pakistan).

## Results

### Impact of (NH_4_)_2_SO_4_ on growth and agronomic parameters of canola plants

The statistical analyses performed on two seasons mean data showed significant differences on all studied features (Tables 3, 5). Results obtained in the current investigation (E1) suggested that (NH_4_)_2_SO_4_ has more positive correlation with respect to plant height, number of branches per plant, fruiting zone length, seed yield per plant, seed yield per hectare and oil percentage compared to NH_4_NO_3_ (Tables 4, 6). Moreover, the effect of (NH_4_)_2_SO_4_ was dose dependent, higher the amount of applied fertilizer, higher value of plant height, number of branches per plant, fruiting zone length, seed yield per plant, seed yield per hectare and oil contents of canola plants were recorded. Correlation analysis was performed in order to evaluate the agronomic characteristics after the (NH_4_)_2_SO_4_ treatment and it was found that significant results have been achieved with plant height (0.998), number of branches per plant (0.953), fruiting zone length (0.987), seed yield per plant (0.994), seed yield per hectare (0.994) (Tables 4, 6).

**Table 3:** Impact of rate and source of nitrogen in the form of ammonium sulfate (NH_4_)_2_SO_4_ and ammonium nitrate NH_4_NO_3_ on the mean yield and yeild attribute during 2017–18.

Treatments	Plant height	No. of branches	Fruiting zone	Seed yield/plant	Seed yield/fed	Oil percentage
Nitrogen						
Ammonium sulfate (kg/fed)						
0	141.5bc*	5.5a*	109.1ac*	29.9ad**	725.7a**	43.02a*
10	150.7b	5.7a	116.4a	32.8a	788.1b	42.5a
20	160.1ab	6.4b	121.7a	35.2b	833.7c	43.5b
30	168.4a	7.1c	131.5b	37.8c	908.2d	43.8b
Ammonium nitrate (kg/fed)						
0	141.2d**	5.3cd*	110.4d**	30.2c**	729.0c**	41.9b*
50	157.1c	6.3bc	124.4c	34.6b	830.4b	45.01a
75	169.9b	7.1b	133.0b	36.1b	861.4b	40.37c
100	194.0a	9a	156.2a	42.4a	1007.2a	38.19c

Value within the column with the same letter are not significantly different (Tukey, HSD; p 0.05), **p 0.01 according to least significant difference (LSD) test.

**Table 4:** Correlation coefficient of nitrogen in the form of ammonium sulfate (NH_4_)_2_SO_4_ and ammonium nitrate NH_4_NO_3_ on the mean yield and yeild attribute during 2017–18.

Correlation	R value	SE	p (r = 0)
Ammonium sulfate			
Plant height	0.998	0.020	0.0000(***)
No. of branches	0.953	0.029	0.0000(***)
Fruiting zone	0.987	0.039	0.0000(***)
Seed yield/plant	0.994	0.034	0.0000(***)
Seed yield/fed	0.994	0.034	0.0000(***)
Oil percentage	0.986	0.051	0.0000(***)
Ammonium nitrate			
Plant height	0.987	0.034	0.0000(***)
No. of branches	0.884	0.076	0.0000(***)
Fruiting zone	0.957	0.045	0.0000(***)
Seed yield/plant	0.953	0.048	0.0000(***)
Seed yield/fed	0.953	0.048	0.0000(***)
Oil percentage	− 0.892	0.071	0.0000(***)

### Impact of NH_4_NO_3_ on growth and agronomic parameters of canola plants

The NH_4_NO_3_ application has considerably influenced the crop’s agronomic and quality traits compared to the control canola plants in the field. The recorded results including the maximum plant height (194 cm), number of branches per plant (9), fruiting zone length (156.2 mm), seed yield per plant (42.4 g) and seed yield per hectare (1007.2 kg) showed an increase in all of the aforementioned agronomic attributes (Tables 3, 5), except for the oil percentage when NH_4_NO_3_ dose increased to maximum (100 kg/ha) (Table 3). A highly positive correlation was also observed between yield attributes and NH_4_NO_3_ rates for plant height (0.987), number of branches per plant (0.887), fruiting zone length (0.957), seed yield per plant (0.953), and seed yield per hectare (0.953), while negative correlation with oil contents was detected (Tables 4, 6).

**Table 5:** Impact of rate and source of nitrogen in the form of ammonium sulfate (NH_4_)_2_SO_4_ and ammonium nitrate NH_4_NO_3_ on the mean yield and yeild attribute during 2018–19.

Treatments	Plant height	No. of branches	Fruiting zone	Seed yield/plant	Seed yield/fed	Oil percentage
Nitrogen						
Ammonium sulfate (kg/fed)						
0	140.2**	5.4a*	107.0b**	30.1d**	717.6a**	41.83b*
10	154.4b	5.9ab	118.1c	33.5c	802.7c	43.1b
20	162.1ab	6.7bc	125.2d	36.0b	862.6b	43.2a
30	172a	7.3c	132.9a	38.8a	929.0d	43.47a
Ammonium nitrate (kg/fed)						
0	138.7a**	5.2cd*	109.3d**	29.8d**	712.9a**	42.51a*
50	155.3c	5.9bc	124c	39.2c	812.3c	41.53ab
75	168.1b	6.7b	132.4b	36.7b	874.2b	40.45b
100	193d	8.9a	153a	41.5a	994.0d	39.54bc

Value within the column with the same letter are not significantly different (Tukey, HSD; p 0.05), **p 0.01 according to least significant difference (LSD) test.

**Table 6:** Correlation coefficient of nitrogen in the form of ammonium sulfate (NH_4_)_2_SO_4_ and ammonium nitrate NH_4_NO_3_ on the mean yield and yeild attribute during 2018–19.

Correlation	R value	SE	p (r = 0)
Ammonium sulfate			
Plant height	0.986	0.041	0.0000 (***)
No. of branches	0.963	0.066	0.0000 (***)
Fruiting zone	0.992	0.046	0.0000 (***)
Seed yield/plant	0.992	0.046	0.0000 (***)
Seed yield/fed	0.976	0.036	0.0000 (***)
Oil percentage	0.939	0.62	0.0000 (***)
Ammonium nitrate			
Plant height	0.985	0.018	0.0000 (***)
No. of branches	0.879	0.066	0.0000 (***)
Fruiting zone	0.967	0.042	0.0000 (***)
Seed yield/plant	0.867	0.078	0.0000 (***)
Seed yield/fed	0.865	0.078	0.0000 (***)
Oil percentage	− 0.273	0.154	0.0867 (ns)

### Effect of foliar application of GA_3_ and K on canola yield and yield components

Application of growth hormone GA_3_ and K caused a significant increase in plant height compared to control plant during a 4-year period (2014–2018). Significant differences were also observed among the treatments (F = 81.913; p ≤ 0.0000, F = 99.79; p ≤ 0.0000, F = 86.782; p ≤ 0.0000, and F = 101.34; p ≤ 0.0000) during growth seasons (Table 7). The maximum plant height was reported with combined treatment of GA_3_ (30 g/ha) and K (6 g/m^2^) (T_10_) followed by T_9_ and T_8_ (Table 7). However, both T_4_ (GA_3_ 0 and K 6.0) and T_8_ (GA_3_ 10 g/ha and K 2.0 g/m^2^) showed an almost insignificant variation in the plant-height measurements compared to other treatments.

**Table 7:** Impact of potassium (K) and gibberellic acid (GA_3_ g/ha) on plant height (cm) of canola during four season 2014–18.

Treatments	Plant height (cm)			
	2014/15	2015/16	2016/17	2017/18
T1 (control)	139.5g	141g	140.5f	140g
T2 (100 g/ha GA_3_)	154.6f	153.8e	155d	154.8e
T3 (150 g/ha GA_3_)	169.4e	169.0d	168.5b	170.0d
T4 (300 g/ha GA_3_)	178.3d	175.2c	177.2c	176.5c
T5 (2.0 cm^−1^ K)	152.1f	150.3f	151.8e	151.9f
T6 (3.5 g/m^2^ K)	159.5b	156.3e	158.5d	159.4e
T7 (6.0 g/m^2^ K)	170.3e	168.9d	169.0b	171.3d
T8 (100 g/ha GA_3_ + 2.0 g/m^2^ K)	177.9c	176.4c	178.4c	176.9c
T9 (150 g/ha GA_3_ + 3.5 g/m^2^ K)	179.5b	180.2b	180.5b	181.0b
T10 (300 g/ha GA_3_ + 6.0 g/m^2^ K)	183.4a	184.1a	184.0a	184.9a
LSD 0.05	4.342	4.201	4.443	4.392
F	81.913	99.79	86.782	101.34
p	0.0000	0.0000	0.0000	0.0000

Value within the column with the same letter are not significantly different (Tukey, HSD; p 0.05), **p 0.01 according to least significant difference (LSD) test.

The foliar application of K and GA_3_ significantly affected the number of branches per canola plant comparing to the control one (T_1_). The highest number of branches per plant were recorded in T_10_ (30GA_3_ g/ha + 6.0 g/m^2^ K) which appeared to have the same trend as that reported for canola plant height (Table 8). A considerable rise in the fruiting zone length (cm) was also observed when combined foliar applications were applied (T_10_). The significant differences among the treatments (F = 101.814; p ≤ 0.0000, F = 123.32; p ≤ 0.0000, F = 126.62; p ≤ 0.0000 and F = 122.4; p ≤ 0.0000) were also recorded for over 4 years of the study (Table 9).

**Table 8:** Impact of potassium (K) and gibberellic acid (GA_3_ g/ha) on Number of branches per plant (cm) of canola during four season 2014–18.

Treatments	Number of branches/plant			
	2014/15	2015/16	2016/17	2017/18
T1 (control)	5.4b	5.2b	5.1f	5.7a
T2 (100 g/ha GA_3_)	5.7bc	5.7b	5.8ef	6.2a
T3 (150 g/ha GA_3_)	7.1ae	7.1ce	7.0cd	7.6dc
T4 (300 g/ha GA_3_)	7.5ef	8.2d	7.6bc	8.7e
T5 (2.0 cm^−1^ K)	6.1c	5.9ae	6.0e	6.4be
T6 (3.5 g/m^2^ K)	6.7a	6.6cd	6.8d	7.1cb
T7 (6.0 g/m^2^ K)	7.0ae	7.2ce	7.1cd	7.7dc
T8 (100 g/ha GA_3_ + 2.0 g/m^2^ K)	7.6ef	7.9de	7.7bc	8.4ad
T9 (150 g/ha GA_3_ + 3.5 g/m^2^ K)	8.3df	8.2d	8.2ab	8.7e
T10 (300 g/ha GA_3_ + 6.0 g/m^2^ K)	8.5d	8.6d	8.7a	8.9e
LSD 0.05	0.503	0.524	0.407	0.556
F	45.761	43.56	45.07	48.097
p	0.0000	0.0000	0.0000	0.0000

Value within the column with the same letter are not significantly different (Tukey, HSD; p 0.05), **p 0.01 according to least significant difference (LSD) test.

**Table 9:** Impact of potassium (K) and gibberellic acid (GA_3_ g/ha) on fruiting zone length (cm) of canola during four season 2014–18.

Treatments	Fruiting zone length (cm)			
	2014/15	2015/16	2016/17	2017/18
T1 (control)	118.0h	120.5d	119.0h	121d
T2 (100 g/ha GA_3_)	123.2g	122.4f	123.2g	123.4f
T3 (150 g/ha GA_3_)	134.5ef	132.3cd	134.0ef	133.4cd
T4 (300 g/ha GA_3_)	140.0cd	139.5bc	140.0cd	138.5bc
T5 (2.0 cm^−1^ K)	121.3g	118.5ef	122.3g	122.5ef
T6 (3.5 g/m^2^ K)	131.0f	128.4d	131.0f	129.4d
T7 (6.0 g/m^2^ K)	136.7de	134.0cd	135.5de	136.0cd
T8 (100 g/ha GA_3_ + 2.0 g/m^2^ K)	143.5bc	141.3bc	141.5bc	143.5bc
T9 (150 g/ha GA_3_ + 3.5 g/m^2^ K)	146ab	145.9ab	148.0ab	146.9ab
T10 (300 g/ha GA_3_ + 6.0 g/m^2^ K)	147.5a	149.0a	147.5a	148.5a
LSD 0.05	3.507	3.231	3.403	3.306
F	101.814	123.32	126.62	122.4
p	0.0000	0.000	0.0000	0.000

Value within the column with the same letter are not significantly different (Tukey, HSD; p 0.05), **p 0.01 according to least significant difference (LSD) test.

Another agronomic trait, number of seeds per plant influenced positively, when foliar applications of K and GA_3_ were applied (individually or combined), it was found that canola plants produce more number of seeds per plant when combined GA_3_ and K were applied (T_10_) during the four seasons of 2014–2018 (Table 10). This parameter seemed to be improved immeasurably in all treatments (T_2_–T_10_) compared to the control plant (T_1_). Therefore, it can be concluded that improvement of this agronomic parameter can be successfully attained with higher dose of the foliarly applied K and GA_3_.

**Table 10:** Impact of potassium (K) and gibberellic acid (GA_3_ g/ha) on number of seeds per plant (g) of canola during four season 2014–18.

Treatments	Number of seed/plant (g)			
	2014/15	2015/16	2016/17	2017/18
T1 (control)	30.2a	31.2a	32.3c	30.3a
T2 (100 g/ha GA_3_)	32.4ef	32.4ae	32.5a	31.5g
T3 (150 g/ha GA_3_)	34.6bc	34.6cd	37.4ef	36.4be
T4 (300 g/ha GA_3_)	36.0bd	36.0bc	38.1dh	37.1cd
T5 (2.0 cm^−1^ K)	33.9abe	33.9ade	34.4a	33.4g
T6 (3.5 g/m^2^ K)	33.5be	33.5de	35.08f	34.08b
T7 (6.0 g/m^2^ K)	36.3cd	36.3bc	37.2dh	36.2cd
T8 (100 g/ha GA_3_ + 2.0 g/m^2^ K)	37.8d	37.8b	38.4bh	37.4fc
T9 (150 g/ha GA_3_ + 3.5 g/m^2^ K)	37.6d	37.6b	39.1bg	38.1fh
T10 (300 g/ha GA_3_ + 6.0 g/m^2^ K)	40.0f	40.0f	41.7g	40.7h
LSD 0.05	1.184	1.172	1.195	1.245
F	50.765	46.486	49.987	51.073
p	0.0000	0.0000	0.0000	0.0000

Value within the column with the same letter are not significantly different (Tukey, HSD; p 0.05), **p 0.01 according to least significant difference (LSD) test.

Since seed yield ha^−1^ is considered as the main interest for canola breeders, and current trial (E2) also showed significant influence of K and GA_3_ on seed yield ha^−1^ (individually or combined) with respect to non-sprayed plants (T_1_), particularly with maximum concentration of K and GA_3_. This important rise in seed yield^−1^ (883.2) was recorded with 30 g/ha GA_3_ and 6 g/m^2^ K foliar application (T_10_). The significant differences were also detected among the following treatments (F = 44.576; p ≤ 0.0000, F = 49.903; p ≤ 0.0000, F = 48.765; p ≤ 0.0000 and F = 51.273; p ≤ 0.0000) applied during the experimental period (Table 11).

**Table 11:** Impact of Potassium (K) and gibberellic acid (GA_3_ g/ha) on seed yield of canola during four season 2014–18.

Treatments	Seed yield			
	2014/15	2015/16	2016/17	2017/18
T1 (control)	685.3e	657.7g	686.3e	667.7g
T2 (100 g/ha GA_3_)	715.0de	684.0fg	716.0de	694.0fg
T3 (150 g/ha GA_3_)	781.0c	762.2de	782.0c	762.2de
T4 (300 g/ha GA_3_)	807.4bc	795.2bcd	808.4bc	795.2bcd
T5 (2.0 cm^−1^ K)	717.2de	729.0ef	718.2de	727.0ef
T6 (3.5 g/m^2^ K)	734.8d	751.4de	735.8d	753.4de
T7 (6.0 g/m^2^ K)	800.8bc	785.6cd	801.8bc	788.6cd
T8 (100 g/ha GA_3_ + 2.0 g/m^2^ K)	827.3	824.8bc	828.3	823.8bc
T9 (150 g/ha GA_3_ + 3.5 g/m^2^ K)	829.5	835.7b	830.5	834.7b
T10 (300 g/ha GA_3_ + 6.0 g/m^2^ K)	878.9a	881.3a	879.9a	883.3a
LSD 0.05	26.724	27.167	25.574	26.543
F	44.576	49.903	48.765	51.273
p	0.0000	0.0000	0.0000	0.0000

Value within the column with the same letter are not significantly different (Tukey, HSD; p 0.05), **p 0.01 according to least significant difference (LSD) test.

The changes in oil percentages, in response to K and GA_3_ application were also investigated. The highest oil percentage was observed at the T_10_ treatment followed by T_3_ and T_5_ during the four cropping seasons of 2014–18 (Table 12).

**Table 12:** Impact of potassium (K) and gibberellic acid (GA_3_ g/ha) on seed oil percentage of canola during four season 2014–18.

Treatments	Seed oil percentage			
	2014/15	2015/16	2016/17	2017/18
T1 (control)	41.09bc	41.65ab	42.36a	42.50c
T2 (100 g/ha GA_3_)	42.53bc	41.53ab	42.35a	43.35c
T3 (150 g/ha GA_3_)	42.96bc	42.94ab	42.89a	43.05c
T4 (300 g/ha GA_3_)	42.51c	41.51b	42.55cda	42.59cbe
T5 (2.0 cm^−1^ K)	42.79bc	42.80ab	42.69cd	42.90be
T6 (3.5 g/m^2^ K)	42.87ab	42.86ac	42.95be	42.99ad
T7 (6.0 g/m^2^ K)	43.17a	43.17c	43.02e	43.08a
T8 (100 g/ha GA_3_ + 2.0 g/m^2^ K)	42.64bc	42.79ab	42.48de	42.60bc
T9 (150 g/ha GA_3_ + 3.5 g/m^2^ K)	42.66bc	42.87ab	42.74bc	42.84de
T10 (300 g/ha GA_3_ + 6.0 g/m^2^ K)	44.56bc	43.85ab	45.54cd	44.69be
LSD 0.05	0.1567	0.1893	0.2101	0.2043
F	7.753	9.873	16.874	17.765
p	0.0000	0.0000	0.0000	0.0000

Value within the column with the same letter are not significantly different (Tukey, HSD; p 0.05), **p 0.01 according to least significant difference (LSD) test.

## Discussion

Application of different forms of fertilizers either in rhizosphere or as foliar application is considered as major agro-inputs as cost effective and increased productivity. For proper care of the health and vigor of canola plants to obtain high yield, a well-maintained fertilization is requisite at certain periods throughout the year. Moreover, nitrogen in that order are major nutrients required for the enhanced yield with appropriate concentration^21^. Further, ammonium sulfate delivers precarious plant nitrogen (N) and sulfur (S) nutrients^22^. The balanced application of S and N is vital with the objective of further improving the canola seeds quality and production^23^. Karamanos et al.^24^ suggested that the optimal ratio of N:S ranging from 7:1 to 5:1 can maximize canola production. In fact, a study conducted by Brennan and M.D.A, 2008 proven that the canola production can be extremely limited in case of sulfur deficiency in soil^25^. The supply of artificial sulfur promotes the nitrogen uptake efficiency of canola production and consequently elevates the level of protein in leaves: this will definitely enhance the crop productivity and yield^26^.

Our results are in agreement with those reported by Chien et al.^28^ in which the plant height and number of branches were boosted when higher rates of ammonium sulfate were applied. Other researchers have reported similar results, indicated that the 1000-seed weight increases proportionately with sulfur and nitrogen levels^28^. Others have suggested that biological yield increases significantly with increasing proportions of nitrogen and sulfur^29^.

Another important factor that must be taken into account is the nutrient deficiency (N) can severely hampers canola productivity^30,31^. Furthermore, the canola yields can be enhanced by a better management of N at the optimum growth stages of canola^2,16^. Nitrogen is an essential plant nutrient that simulates its meristematic activity, cell elongation, and elevates the photosynthesis of canola. These factors will ultimately boost growth and yield of the canola plant^32^. A pervious study published by Khan et al.^16^, they haves demonstrated that 3.8 qt/ha (Quintal/hectare) oil yield was achieved through rigorous application of 60 kg of nitrogen per hectare. Similar findings have been made in other studies highlighting the importance of nitrogen supplementation in the refinement of the rapeseed yields in diverse agro-climatic conditions^33^.

As far as current data suggested, N has strongly and significantly correlated with the seed yield per hectare, plant height, number of branches per plant, fruiting zone length, and number of seed per plant, in addition to the enhancement of the number of pods per seed, 1000 seed weight, biological yield, seed yield, and oil yield^29,33^. On this basis, it can be concluded that the canola production depends on the selection of the correct dose, source, and timing of nitrogen fertilizer application. Unbalanced application of nitrogen fertilizer may adversely affect the canola production^6^. The source of N fertilizer may also change the plant N uptake and soil N availability and hence impacting the ultimate canola productivity^34^. In our experiments, two sources of N were tested and compared one with another. The subsequent results showed that ammonium sulfate had significantly contributed to the enhancement of canola production comparing to the ammonium nitrate^35^. However, it has been reported that the application of ammonium sulfate reduces the pH of the soil as well as dissolution of many other nutrients resulting in negative impacts on plant growth and development compared to ammonium nitrate^36^. Based on N management concept, it is well accepted fact that ammonium-based fertilizers are issue to ammonia (NH_3_) volatilization in soils with pH > 7, but this has been ignored in choice of making on S fertilization. The influence of various treatments related to the application of GA_3_ and K fertilizers were also studied in accordance with the yield parameters of canola. The results of the present study provide evidence that all the agronomic traits and oil percentage tend to increase with increasing levels of foliar application of K and GA_3_ solely or in combined form compared to the unsprayed plants. A significant increase was recorded using different treatments of GA_3_ and K in plant height, number of branches per plant, fruiting zone length, seed yield per plant, seed yield/ha and seed oil percentage compared to control (T_1_–T_10_).

In view of the aforementioned findings, it can be concluded that combined form of GA_3_ and K (T_10_) presents a potential strategy to enhance growth performance of canola. The promoting effect of GA_3_ and K treatments contribute to the metabolic and other physiological processes leading to better crop yields. Interestingly, for the majority of the studied traits, the K application (T_4_) acts similarly and almost insignificantly to the combined application (T_8_) of K (3.5 g/m^2^) and GA_3_ (15 g/ha), this could be attributed to the key role of K in improving canola yields (Tables 7, 8, 9, 10, 11, 12).

GA_3_ and K fertilizer application is necessary to increase the vegetative and reproductive growth of canola plant^37^. These fertilizers could be involved in improving defence mechanisms of canola plant which may consequently affect the seed yield. Similar results have been reported using these same treatments on sesame plant^30^. Likewise, foliar application of potassium and gibberellic acid alone or in combination increases the plant vegetative and reproductive growth of the plant resulting in the enhancement of the yield per unit^38^. In fact, in a study reported by Imran and Khan^39^, the application of K fertilizer not only enhances the yield per unit, fresh nut and kernel dry mass (splitting percentage), it also reduces the blank percentage. It was also observed that in absence of gibberellic acid applications, the blank percentage and splitting percentage could be ameliorated^40^.

Jan et al.^41^ reported that high concentrations of potassium K and Zing Zn after the simultaneous foliar applications of GA_3_ and K separately or in combination could be found in canola plant leaves. The evidences of this study suggest that the interactive effects of GA_3_ and K can be employed in the aim of improving morphological aspects and yield attributes of canola. It can also be expected that these interactive effects may elevate the plant resistance against various biotic and abiotic stresses, carbohydrate translocation, and the photosynthesis process^40^. Khan et al. (2019) also mentioned that these fertilizers (GA_3_ and K) might strengthen the defence mechanism of the plant which ultimately impacts the plant growth and yield^42,43^. In short, with appropriate application of N fertilizers, GA_3_, and K, canola yields can be substantially improved.

## Conclusion

Nitrogen is an essential nutrient for the metabolic function and production process of the canola plant or any other plant. Therefore, the canola yields can be monitored with the application of N fertilizers pertaining to different sources and proportions. The optimum levels of N fertilizer were found to be 30 kg/ha ammonium sulfate and 100 kg/ha ammonium nitrate. These data have been obtained according to the agronomic yields of a 4-year study (2014–18). Another fact to consider is that Ammonium nitrate (NH_4_NO_3_) is more efficient and readily available source of nitrogen compared to ammonium sulfate [(NH4)_2_NO_3_]. This study has recommended the optimum value and source in subtropical region of the world. On the contrary, gibberellic acid and potassium influence the plant growth and its development, enable the plant to survive in nutrient deficient soil and increase the yield in the four growing seasons (2014–18). It is suggested that canola plant illustrated maximum potential of yield at high dose of GA_3_ (30 g/ha) and K (6.0 g/m^2^) alone or in combination.
